# Enhanced marine sulphur emissions offset global warming and impact rainfall

**DOI:** 10.1038/srep13055

**Published:** 2015-08-21

**Authors:** B. S. Grandey, C. Wang

**Affiliations:** 1 Center for Environmental Sensing and Modeling, Singapore-MIT Alliance for Research and Technology, Singapore; 2 Center for Global Change Science, Massachusetts Institute of Technology, Cambridge, Massachusetts, USA

## Abstract

Artificial fertilisation of the ocean has been proposed as a possible geoengineering method for removing carbon dioxide from the atmosphere. The associated increase in marine primary productivity may lead to an increase in emissions of dimethyl sulphide (DMS), the primary source of sulphate aerosol over remote ocean regions, potentially causing direct and cloud-related indirect aerosol effects on climate. This pathway from ocean fertilisation to aerosol induced cooling of the climate may provide a basis for solar radiation management (SRM) geoengineering. In this study, we investigate the transient climate impacts of two emissions scenarios: an RCP4.5 (Representative Concentration Pathway 4.5) control; and an idealised scenario, based on RCP4.5, in which DMS emissions are substantially enhanced over ocean areas. We use mini-ensembles of a coupled atmosphere-ocean configuration of CESM1(CAM5) (Community Earth System Model version 1, with the Community Atmosphere Model version 5). We find that the cooling effect associated with enhanced DMS emissions beneficially offsets greenhouse gas induced warming across most of the world. However, the rainfall response may adversely affect water resources, potentially impacting human livelihoods. These results demonstrate that changes in marine phytoplankton activity may lead to a mixture of positive and negative impacts on the climate.

DMS is a product of dimethylsulfoniopropionate produced by many species of phytoplankton^1^. Much of the DMS emitted to the atmosphere is oxidised to sulphur dioxide then to sulphuric acid to form sulphate aerosol. Sulphate aerosol impacts climate via direct^2^ and indirect^3^,^4^ effects on radiation and clouds. DMS is the primary source of sulphate aerosol over remote ocean regions, which cover much of the Earth’s surface. Hence DMS plays an important role in the Earth’s climate system.

Future DMS emissions are uncertain. There is evidence to suggest that significant regional variations in marine primary productivity may have occurred during the twenty-first century^5^. The reasons for these variations remain unclear and may be due to natural variability. Since the climatic impact of DMS emissions is likely to be strongly dependent on the regional distribution of the DMS emissions^6^, such regional variability may have important implications for climate.

Furthermore, feedbacks between future climate change and DMS emissions might exist^7^. It has been suggested that increasing atmospheric greenhouse gas concentrations may enhance DMS emissions, and thus sulphate aerosol concentrations, in the Southern Ocean, introducing a negative feedback to offset the warming^8^,^9^. Increasing ocean acidification by carbon dioxide may also impact DMS emissions^10^,^11^. The net effect of these feedbacks to future climate change has yet to be revealed.

Alongside natural variation and feedback processes, it is possible that DMS emissions may be directly affected by anthropogenic activities. For example, ocean fertilisation has been proposed as a possible geoengineering method to remove carbon dioxide from the atmosphere in order to mitigate climate change^12^. A possible side effect of such fertilisation is an enhancement of DMS emissions^13^. The associated radiative effects may have a large cooling effect even if only a small percentage of the total ocean area is fertilised^14^. It has been suggested that targeted fertilisation of specific regions may be a possible method to offset some of the climatic changes associated with increasing greenhouse gas concentrations, such as changes in the Southern Hemisphere winds^15^. Therefore, artificial ocean fertilisation may form a basis not only for carbon dioxide removal (CDR)^16^, but also for solar radiation management (SRM)^17^.

An interesting question to ask is: if DMS emissions were to be substantially increased, what would be the effect on surface temperature and precipitation? To answer this question, we need to account not only for local processes but also for dynamical processes occurring on regional and even larger scales. For this reason, we use a coupled atmosphere-ocean aerosol-climate model to investigate two emissions scenarios for the twenty-first century:

1. An RCP4.5 (Representative Concentration Pathway 4.5) control^18^. This is a commonly used future projection scenario of greenhouse gas concentrations, aerosol (including aerosol precursor) emissions, and land use change. RCP4.5, a stabilisation scenario, assumes substantially lower carbon dioxide emissions than RCP8.5, a “highly energy-intensive scenario”^19^.
2. “EnDMS”, an experimental scenario with enhanced DMS emissions (Fig. 1). Following idealised assumptions about nutrient limitation and the other factors affecting DMS emissions (see Methods), EnDMS can be interpreted as an idealised upper-bound scenario in which ocean fertilisation prevents nutrient limitation of DMS emissions.

Non-DMS aerosol emissions and greenhouse gas concentrations follow RCP4.5 in both scenarios. Hence, the impact of increased marine primary productivity on the carbon cycle is not considered. We have chosen to focus on prescribed DMS emissions in this study, in order to simplify physical interpretation of the results. To account for internal variability, we have performed a three-member mini-ensemble for each of the two emissions scenarios.

## Results and Discussion

In the RCP4.5 ensemble, the global mean surface temperature increases by over 2 °C over the twenty-first century (Fig. 2a), primarily due to increased greenhouse gas induced warming. Over land, the mean surface warming is approximately 3 °C (Fig. 2b). On average, the oceans warm more slowly than land, partly due to the mixing of heat into the ocean, and partly due to other feedbacks^20^. The warming is spatially inhomogeneous (Fig. 3a,d). The largest warming occurs over the Arctic, due to surface albedo and temperature feedbacks^21^. Cooling, as opposed to warming, occurs over part of the North Atlantic ocean, south of Greenland^22^.

The EnDMS ensemble also produces a global mean warming trend (Fig. 2a). However, compared to RCP4.5, the EnDMS warming is delayed by approximately five decades at the end of the century. The delay occurs because a strengthened negative radiative flux perturbation (RFP, Table S2) leads to a widespread cooling in the EnDMS ensemble compared to the RCP4.5 ensemble (Fig. 3c,f). In particular, the enhanced DMS emissions exert a strong radiative effect of over 2 W/m^2^ via low and mid-level clouds in the Southern Hemisphere mid-latitudes and Northern Hemisphere high-latitudes (Fig. S1). Global warming is beneficially offset across most of the world: when RCP4.5 averaged across 2010–2029 is used as a reference (Ref), the EnDMS−Ref differences are almost always smaller than the RCP4.5−Ref differences during both 2040–2059 (Fig. 3b) and 2080–2099 (Fig. 3e).

Global mean precipitation increases in the RCP4.5 ensemble (Fig. S2). The regional response is highly inhomogeneous (Fig. 4a,d), although a general pattern can be observed: precipitation increases across much of the tropics and high latitudes, but decreases across much of the subtropics. The global mean increase, and much of the large-scale response, is likely related to greenhouse gas induced warming^23^. However, the increase in precipitation over East Asia is likely to be primarily due to reductions in anthropogenic aerosol emissions.

The enhanced DMS emissions in EnDMS act to reduce precipitation across most of the world (Fig. 4c,f). A component of this reduction can be explained by a fast response^24^,^25^ (Fig. S3) associated with the fast response of the latent heat flux at the surface (Table S3), combined with a slow response associated with the sea surface temperature (SST) response. Over much of the subtropics, the DMS induced reduction of precipitation combines additively with the greenhouse gas induced reduction of precipitation. This contributes to a highly inhomogeneous precipitation response in the EnDMS ensemble average. Over much of the subtropics, the EnDMS−Ref differences are larger in magnitude than the RCP4.5−Ref differences during both 2040–2059 (Fig. 4b) and 2080–2099 (Fig. 4e). Reductions in precipitation over Europe, the Horn of Africa, and Pakistan (Fig. 4b) may have an adverse impact on the environment and human livelihoods.

## Conclusions

Our model results demonstrate that enhanced DMS emissions may impact climate. A large increase in DMS emissions might beneficially offset global warming across the world. For the simulations considered here, the largest surface temperature offset is found in the Arctic, the region which is most susceptible to global warming.

A large increase in DMS emissions might also act to offset an increasing trend in the global mean precipitation. However, regional inhomogeneities in the precipitation response should also be considered. For example, enhanced DMS emissions might lead to a substantial reduction in precipitation over Europe, adversely impacting the hydrological cycle, the environment, and human livelihoods.

We have not explored the feasibility of artificially increasing DMS emissions via ocean fertilisation, either as a deliberate solar radiation management (SRM) technique or as side effect of carbon dioxide removal (CDR) efforts. It would be interesting to investigate more realistic fertilisation scenarios using models with more detailed biogeochemical processes and feedbacks, such as the ones which have been used to investigate how DMS emissions may respond to increasing carbon dioxide concentrations^9^,^11^. We invite other researchers with more expertise in marine biogeochemical modelling to explore this further.

Even if it were possible to artificially increase DMS emissions, we would not necessarily recommend such a course of action. In addition to potentially impacting the hydrological cycle and human livelihoods, ocean fertilisation may present many other dangers that we have not explored here, such as the impact on marine ecosystems. If ocean fertilisation is to be seriously considered as a possible geoengineering method to partially offset greenhouse gas induced warming, more research is required.

## Methods

### Emissions Scenarios

#### RCP4.5

**Figure 1:**
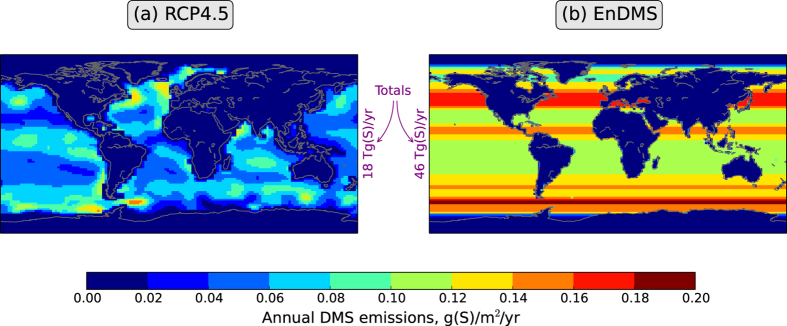
Annual DMS emissions for the (**a**) RCP4.5 and (**b**) EnDMS scenarios. Global totals are provided at the side of each map. The mass unit “g(S)” refers to grams of sulphur. For each latitude band and month, the oceanic DMS emissions in EnDMS are equal to the maximum found in RCP4.5. Since the EnDMS emissions are constructed for each month separately, the annual DMS emissions in EnDMS can be greater than the annual RCP4.5 maximum for any given latitude. The figure was created using Python.

**Figure 2:**
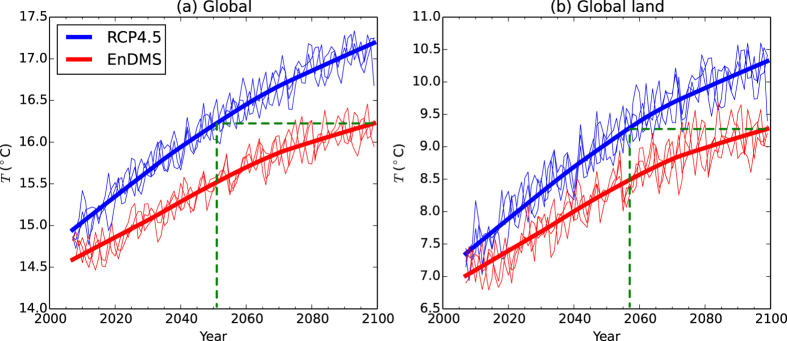
Time series of annual mean radiative surface temperature (*T*) for the RCP4.5 and EnDMS ensembles. (**a**) Global (land and ocean) area-weighted annual mean *T*. (**b**) Global land-only area-weighted annual mean *T*, using a land fraction threshold of 0.9. The thinner blue lines show annual means for each simulation in the RCP4.5 three-member mini-ensemble. The thicker blue lines show robust locally weighted regression smoothing (LOESS) curves^37^,^38^, which have been calculated using a smoothing parameter of *f* = 0.6 and three iterations of fitting, using the RCP4.5 ensemble mean as input. The red lines correspond to the EnDMS ensemble. The green dashed lines illustrate the lag between the RCP4.5 and the EnDMS LOESS curves at the end of the twenty-first century. Years are defined to start in December, so that the December-January-February season is not divided across different annual means. Hence, when calculating annual means for any given year, data from December in the given year are excluded while data from the previous year are included.

**Figure 3:**
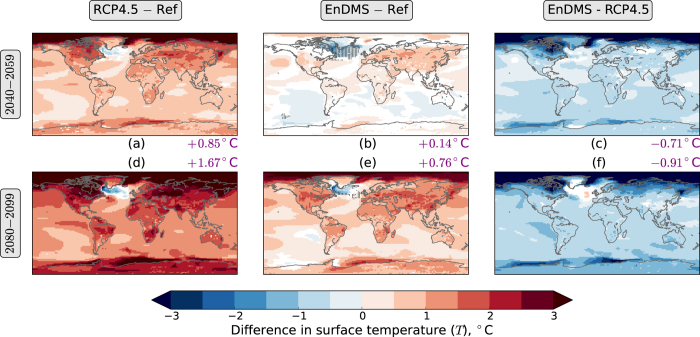
Differences in annual mean radiative surface temperature (*T*). (**a**) RCP4.5 averaged across 2040–2059—Ref (the reference, RCP4.5 averaged across 2010–2029). (**b**) EnDMS averaged across 2040–2059—Ref. (**c**) EnDMS—RCP4.5, averaged across 2040–2059. (**d**) RCP4.5 averaged across 2080–2099—Ref. (**e**) EnDMS averaged across 2080–2099—Ref. (**f**) EnDMS—RCP4.5, averaged across 2080–2099. Years are defined to start in December. Ensemble means are used. Area-weighted mean differences are shown in purple text at the side of each map. White indicates locations where the differences do not have the same sign for all three sets of initial conditions. Stippling in (**b**,**e**) indicates locations where the EnDMS−Ref difference is larger in magnitude than the RCP4.5−Ref difference for that period. The figure was created using Python.

**Figure 4:**
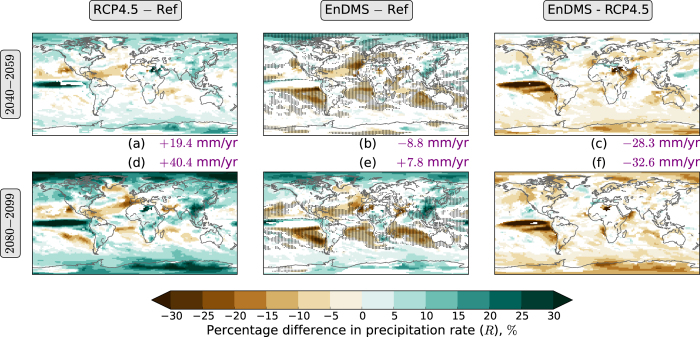
Percentage differences in annual precipitation rate (*R*), relative to Ref (the reference, the RCP4.5 mini-ensemble averaged across 2010–2029). (**a**) RCP4.5 averaged across 2040–2059—Ref. (**b**) EnDMS averaged across 2040–2059—Ref. (**c**) EnDMS−RCP4.5, averaged across 2040–2059. (**d**) RCP4.5 averaged across 2080–2099—Ref. (**e**) EnDMS averaged across 2080–2099—Ref. (**f**) EnDMS−RCP4.5, averaged across 2080–2099. Years are defined to start in December. Ensemble means are used. Area-weighted mean differences are shown in purple text at the side of each map. White indicates locations where the differences do not have the same sign for all three sets of initial conditions. Stippling in (**b**,**e**) indicates locations where the EnDMS−Ref difference is larger in magnitude than the RCP4.5−Ref difference for that period. The figure was created using Python.

For the RCP4.5 control simulations, land use, greenhouse gas concentrations, and aerosol emissions follow the RCP4.5 future projection scenario^18^. AeroCom DMS emissions are used^26^. These DMS emissions vary between different months but are invariant across different years. The total annual DMS emissions are shown in Fig. 1a. The global total is 18 Tg(sulphur)/yr, which accounts for 22% of global sulphur emissions in year-2000 (Table S1). This is lower than an alternative DMS climatology, which estimates the global total to be 28 Tg(sulphur)/yr^27^. It is worth noting that not all of the DMS is oxidised to sulphur dioxide^28^.

#### EnDMS

In the “EnDMS” (enhanced DMS) scenario, greenhouse gas concentrations, emissions of non-DMS aerosol species, and land-use change are the same as in RCP4.5. However, for each latitude band, monthly oceanic DMS emissions are changed to the maximum AeroCom DMS emissions for that latitude and month. For model grid boxes with both ocean and land, these values are scaled by the ocean fraction of the grid box. The total annual DMS emissions are shown in Fig. 1b. The global total is 46 Tg(sulphur)/yr, more than 2.5 times as much as in the RCP4.5 simulation. However, the increase between RCP4.5 and EnDMS is smaller than the decreasing trend in anthropogenic sulphur dioxide emissions between year-2000 and year-2080 (Table S1).

An analysis of the feasibility of the EnDMS scenario is outside the scope of this paper. However, it is worth noting that if the entire Southern Ocean region “were to respond as observed during SOFeX [the Southern Ocean Iron Enrichment Experiment], the additional flux of DMS would result in a total emission of 14 Tg of S per year”^13^. Hence the EnDMS scenario, which assumes a global increase of 28 Tg(S)/yr, may not be beyond the realm of possibility. However, EnDMS is much more extreme than the scenario of a “20 percent increase in DMS production integrated over the entire [Southern Ocean]”^14^ considered by Wingenter *et al*. (2007).

In order to interpret the EnDMS scenario, it is helpful to consider the factors which influence DMS emissions. Marine primary productivity depends on factors such as temperature and light, although the availability of nutrients such as iron and nitrogen often appears to be a limiting factor^29^. In addition to depending on marine primary productivity, ocean surface concentrations of DMS depend on other factors such as phytoplankton species composition^1^ and pH^11^. (It is worth noting that only a weak correlation exists between DMS concentration and chlorophyll concentration, a commonly used indicator of ocean productivity^30^.) For a given DMS surface concentration, the flux to the atmosphere is further influenced by wind speed.

In constructing the EnDMS scenario, two assumptions are made:

1. For each latitude band and month, the DMS emissions are not nutrient-limited in at least one grid box of the AeroCom data set.
2. For each latitude band and month, all other factors affecting DMS emissions (temperature, light, species composition, pH, and wind speed) are approximately invariant with respect to both longitude and year.

Based on these assumptions, EnDMS can be interpreted as an idealised upper-bound scenario in which large-scale ocean fertilisation prevents nutrient limitation of DMS emissions from occurring in any part of the ocean. Such fertilisation may involve multiple nutrients: for example, if only iron fertilisation were to be carried out, then other nutrients may become the limiting factor. In reality, the situation is likely to be much more complicated than this. For example, fertilisation may lead to changes in the phytoplankton species composition in any given location.

### Model Configuration

The scenarios are investigated using a coupled atmosphere-ocean configuration of CESM1(CAM5)^31^. CESM1.0.4 (Community Earth System Model version 1.0.4) is similar to CCSM4 (Community Climate System Model version 4)^32^. CAM5 (Community Atmosphere Model version 5)^33^, the atmospheric model used in this study, contains a modal aerosol model with three lognormal modes (MAM3)^28^. Aerosol indirect effects are represented in the stratiform cloud microphysics scheme^34^,^35^. The CESM1(CAM5) configuration used here is similar to the “B_1850-2000_CAM5_CN” component set, reconfigured to handle projections of future scenarios.

The atmosphere and land models are run on a finite volume grid with a horizontal resolution of 1.9° × 2.5°. The atmosphere has 30 levels in the vertical. A gx1v6 displaced dipole grid, which has a horizontal resolution of approximately 1° × 1°, is used for the ocean and sea ice models.

Initial conditions from twentieth century simulations are used to initialise the simulations in year-2006. Using the three different sets of initial conditions, a mini-ensemble of three simulations is performed for each scenario. The simulations finish in December 2099.

### Radiative Flux Perturbations

Radiative flux perturbation (RFP) values are calculated and decomposed^36^ using prescribed-SST simulations. These simulations are described in the caption of Table S2. They are based on the “F_2000_CAM5” component set. The atmosphere and land grid is the same as that used in the transient atmosphere-ocean simulations. These prescribed-SST simulations are also used to diagnose the fast response^24^ of the surface energy budget and precipitation (Table S3).

The CESM(CAM5) data analysed in this paper are available via figshare: http://dx.doi.org/10.6084/m9.figshare.1483372

## Additional Information

**How to cite this article**: Grandey, B. S. and Wang, C. Enhanced marine sulphur emissions offset global warming and impact rainfall. *Sci. Rep*. **5**, 13055; doi: 10.1038/srep13055 (2015).

## Supplementary Material

Supplementary Information
