# Supplementary material for: Enhanced marine sulphur emissions offset global warming and impact rainfall
Source: Sci Rep. 2015 Aug 21;5:13055. doi: 10.1038/srep13055 (PMC4543957; doi:10.1038/srep13055)
Supplement: Supplementary Information [file srep13055-s1.pdf]

Supporting Tables and Figures for *Enhanced marine sulphur emissions offset global warming and impact rainfall*

B. S. Grandey*[1] and C. Wang[1,2]

[1]Center for Environmental Sensing and Modeling, Singapore-MIT Alliance for Research and Technology, Singapore.
[2]Center for Global Change Science, Massachusetts Institute of Technology, Cambridge, Massachusetts, USA.

April 30, 2015

Data analysis and figure production

As in the main body of the manuscript, data analysis was performed and figures were produced using Climate Data Operators and a number of Python packages (including Numpy, Scientific, Scipy, Bio, Matplotlib, and the Matplotlib Basemap Toolkit).

References

[1] Liu, X. *et al.* Toward a minimal representation of aerosols in climate models: description and evaluation in the Community Atmosphere Model CAM5. *Geoscientific Model Development* **5**, 709–739 (2012). URL http://www.geosci-model-dev.net/5/709/2012/.

[2] Ghan, S. J. Technical Note: Estimating aerosol effects on cloud radiative forcing. *Atmospheric Chemistry and Physics* **13**, 9971–9974 (2013). URL http://www.atmos-chem-phys.net/13/9971/2013/.

[3] Ghan, S. J. *et al.* Toward a Minimal Representation of Aerosols in Climate Models: Comparative Decomposition of Aerosol Direct, Semidirect, and Indirect Radiative Forcing. *Journal of Climate* **25**, 6461–6476 (2012). URL http://journals.ametsoc.org/doi/abs/10.1175/JCLI-D-11-00650.1.

*benjamin@smart.mit.edu

Table S1: **Sulphur emissions in year-2000 and year-2080 for the two scenarios.** The anthropogenic sulphur dioxide (SO_2) emissions category includes biomass burning emissions. It is assumed that 2.5% of the SO_2 emissions are emitted as primary sulphate. The mass unit "g(S)" refers to grams of sulphur. The DMS emissions are not directly comparable to the SO_2 emissions, due to the fact that not all of the DMS is oxidised to SO_2. Using MAM3, the aerosol model used in this study, Liu et al. (2012)[1] found that for DMS emissions of 18 Tg(S)/yr approximately 15 Tg(S)/yr is oxidised to SO_2.

Sulphur emissions in Tg(S)/yr	Year-2000 emissions RCP4.5	Year-2000 emissions EnDMS	Year-2080 emissions RCP4.5	Year-2080 emissions EnDMS
Total	84.6	112.5	43.5	71.4
Anthropogenic SO_2	53.9	53.9	12.8	12.8
Continuous volcanic SO_2	12.5	12.5	12.5	12.5
DMS	18.2	46.1	18.2	46.1
DMS as % of total	22%	41%	42%	65%

Table S2: **Top-of-atmosphere (TOA) radiate flux perturbation (RFP) values, calculated from prescribed-SST simulations.** The sea-surface temperatures (SSTs) and greenhouse gas concentrations have been prescribed using year-2000 climatological values. The prescribed-SST simulations differ only in their aerosol (including aerosol precursor) emissions: (i) year-1850; (ii) year-2000 for RCP4.5; (iii) year-2000 for EnDMS; (iv) year-2080 for RCP4.5; and (v) year-2080 for EnDMS. The five simulations have each been run for 23 years. The first two years and eleven months have been treated as spin-up, while the remaining twenty years have been used to calculate the RFP values in the table. The RFP values have been decomposed using the method recommended by Ghan (2013)[2], which relies on two calls to the radiation scheme: the first call includes direct aerosol–radiation interaction; the second call excludes direct aerosol–radiation interaction, allowing diagnosis of "clean-sky" fluxes. The decomposed RFPs for the year-2000 RCP4.5 aerosol emissions are in approximate agreement with Ghan et al. (2012)[3]. Zonal mean distributions of the net cloud RFP values are shown in Fig. S1.

Radiative flux perturbation (RFP, W/m^2), relative to year-1850 aerosol emissions	Year-2000 aerosol emissions RCP4.5	Year-2000 aerosol emissions EnDMS	Year-2000 aerosol emissions EnDMS − RCP4.5	Year-2080 aerosol emissions RCP4.5	Year-2080 aerosol emissions EnDMS	Year-2080 aerosol emissions EnDMS − RCP4.5
Direct radiative effect	-0.01	-0.07	-0.07	+0.03	-0.03	-0.07
Surface albedo (clean-sky)	+0.01	-0.10	-0.12	-0.02	-0.07	-0.05
SW cloud (clean-sky)	-2.12	-3.96	-1.84	-0.57	-2.80	-2.23
LW cloud	+0.59	+1.33	+0.74	+0.28	+1.14	+0.86
Total	-1.52	-2.80	-1.28	-0.27	-1.76	-1.49

Table S3: **Fast response of surface temperature, surface energy balance, and precipitation, calculated from prescribed-SST simulations.** The sea-surface temperatures (SSTs) and greenhouse gas concentrations have been prescribed using year-2000 climatological values. The prescribed-SST simulation differ only in their aerosol (including aerosol precursor) emissions (see Table S2). The regional distribution of the fast response of precipitation is shown in Fig. S3.

	Year-2000 aerosol emissions EnDMS−RCP4.5	Year-2080 aerosol emissions EnDMS−RCP4.5
Surface net SW flux (downward; W/m^2)	-2.22	-2.55
Surface net LW flux (upward; W/m^2)	-0.57	-0.71
Surface sensible heat flux (W/m^2)	-0.00	-0.03
Surface latent heat flux (W/m^2)	-0.36	-0.37
Surface latent heat flux (% rel. to RCP4.5)	-0.42 %	-0.43 %
Precipitation rate (mm/yr)	-4.49	-4.60
Precipitation rate (% rel. to RCP4.5)	-0.42 %	-0.43 %

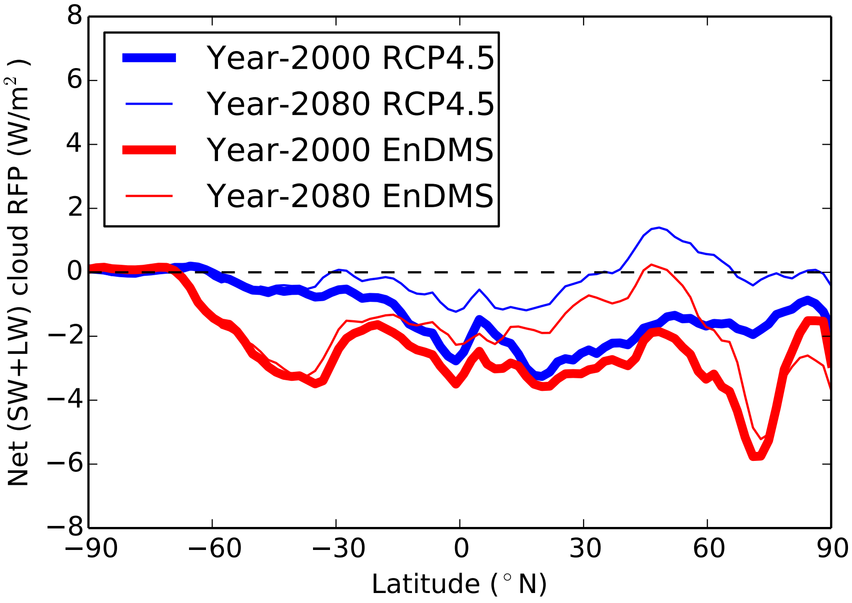

Figure S1: **Zonal mean net cloud RFP values, calculated from prescribed-SST simulations.** These cloud RFPs include both indirect effects and semi-direct effects. The RFP calculation methodology, which follows Ghan (2013) [2], is briefly described in the caption of Table S1. The RFPs are calculated relative to year-1850 aerosol (and aerosol precursor) emissions.

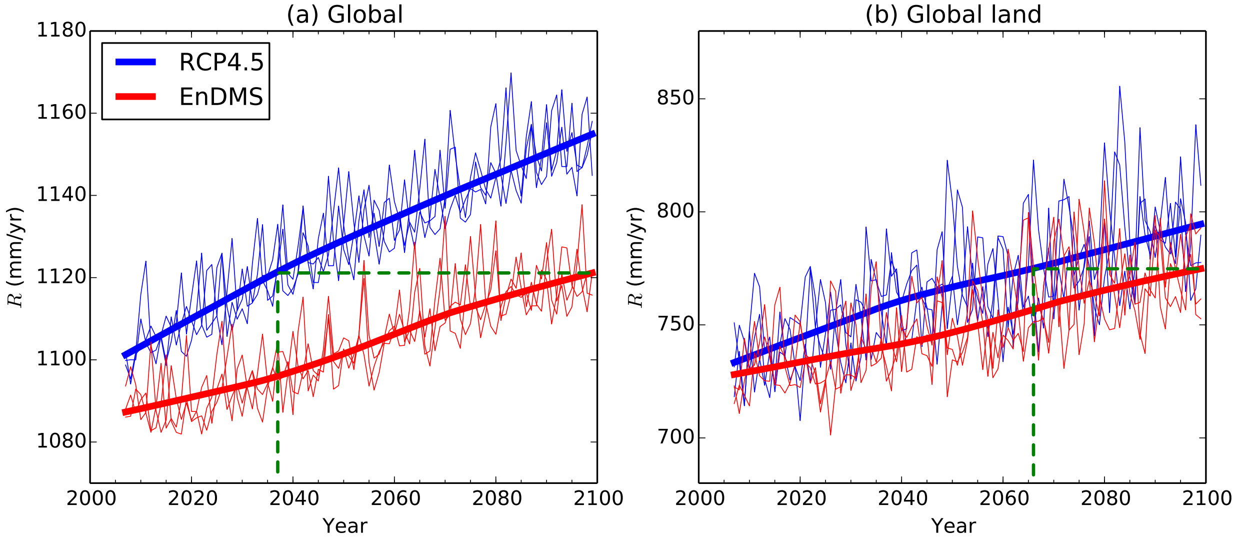

Figure S2: Time series of annual precipitation rate (R) for the RCP4.5 and EnDMS ensembles. (a) Global (land and ocean) area-weighted annual mean R. (b) Global land-only area-weighted annual mean R, using a land fraction threshold of 0.9. The thinner blue lines show annual means for each simulation in the RCP4.5 three-member ensemble. The thicker blue lines show robust locally weighted regression smoothing (LOESS) curves, which have been calculated using a smoothing parameter of $f = 0.6$ and three iterations of fitting, using the RCP4.5 ensemble mean as input. The red lines correspond to the EnDMS ensemble. The green dashed lines illustrate the lag between the RCP4.5 and the EnDMS LOESS curves at the end of the twenty-first century. Years are defined to start in December, so that the December-January-February season is not divided across different annual means. Hence, when calculating annual means for any given year, data from December in the given year are excluded while data from the previous year are included.

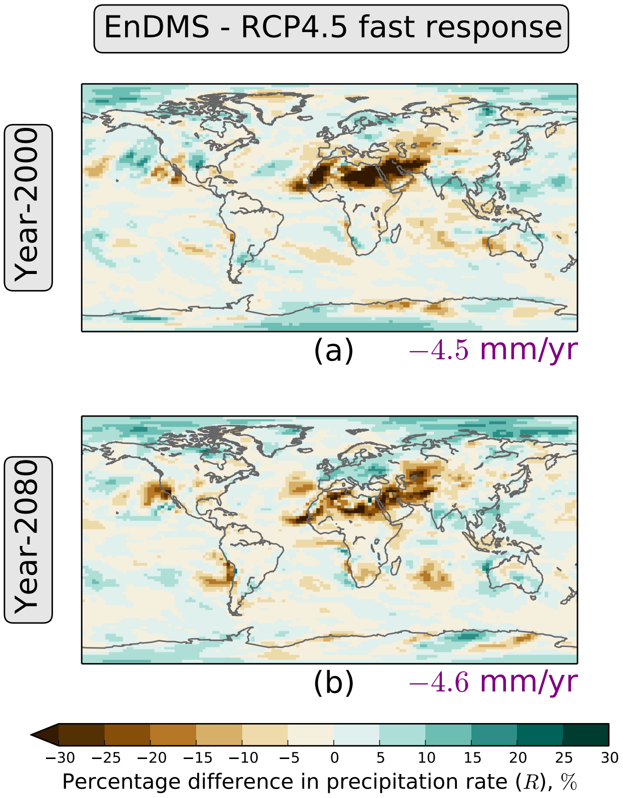

Figure S3: **Fast response of the annual precipitation rate (R), calculated from the prescribed-SST simulations.** (a) Year-2000 aerosol emissions. (b) Year-2080 aerosol emissions. The prescribed-SST simulations are described in Table S2. Area-weighted mean differences are shown in purple text under each map. The figure was created using Python.
